# Progressive Attention-Enhanced EfficientNet–UNet for Robust Water-Body Mapping from Satellite Imagery

**DOI:** 10.3390/s26030963

**Published:** 2026-02-02

**Authors:** Mohamed Ezz, Alaa S. Alaerjan, Ayman Mohamed Mostafa, Noureldin Laban, Hind H. Zeyada

**Affiliations:** 1 Computer Science Department, College of Computer and Information Sciences, Jouf University, Sakaka 72388, Saudi Arabia; maismail@ju.edu.sa; 2 Information Systems Department, College of Computer and Information Sciences, Jouf University, Sakaka 72388, Saudi Arabia; 3 Data Reception, Analysis and Receiving Station Affairs, National Authority for Remote Sensing & Space Sciences, Cairo 1564, Egypt; nourlaban@narss.sci.eg (N.L.); hind.zyada@narss.sci.eg (H.H.Z.)

**Keywords:** water-body delineation, remote sensing imagery, deep learning architecture, modified EfficientNet–UNet, convolutional block attention module (CBAM), attention mechanisms

## Abstract

The sustainable management of water resources and the development of climate-resilient infrastructure depend on the precise identification of water bodies in satellite imagery. This paper presents a novel deep learning architecture that integrates a convolutional block attention module (CBAM) into a modified EfficientNet–UNet backbone. This integration allows the model to prioritize informative features and spatial areas. The model robustness is ensured through a rigorous training regimen featuring five-fold cross-validation, dynamic test-time augmentation, and optimization with the Lovász loss function. The final model achieved the following values on the independent test set: precision = 90.67%, sensitivity = 86.96%, specificity = 96.18%, accuracy = 93.42%, Dice score = 88.78%, and IoU = 79.82%. These results demonstrate improvement over conventional segmentation pipelines, highlighting the effectiveness of attention mechanisms in extracting complex water-body patterns and boundaries. The key contributions of this paper include the following: (i) adaptation of CBAM within a UNet-style architecture tailored for remote sensing water-body extraction; (ii) a rigorous ablation study detailing the incremental impact of decoder complexity, attention integration, and loss function choice; and (iii) validation of a high-fidelity, computationally efficient model ready for deployment in large-scale water-resource and ecosystem-monitoring systems. Our findings show that attention-guided segmentation networks provide a robust pathway toward high-fidelity and sustainable water-body mapping.

## 1. Introduction

The accurate and reliable mapping of surface water bodies from remote sensing imagery remains a fundamental requirement for sustainable environmental management, flood monitoring, and climate change assessment. Surface water represents a vital component of the Earth’s hydrological system and plays a crucial role in sustaining ecological balance, supporting biodiversity, and regulating natural water cycles. As presented in [1,2,3,4,5], surface water dynamics significantly influence climate regulation, agricultural productivity, and urban planning. Therefore, the accurate identification and monitoring of surface water are essential for effective water resource management, environmental protection, and sustainable socio-economic development. The authors of [6,7,8,9,10] emphasize that the precise delineation of water bodies is fundamental to flood assessment, drought monitoring, and long-term watershed planning.

Segmentation through image processing techniques remains one of the most widely used approaches for extracting water bodies from digitally available satellite imagery. In this context, segmentation assigns labels to pixels based on their distinct spectral or spatial characteristics, enabling the separation of water regions from the surrounding land. As explained in [11], semantic segmentation analyzes pixel-level properties across different regions of an image and classifies previously unseen areas using either rule-based algorithms or machine learning-based models.

Satellite imaging sensors are generally divided into two major classes. According to the display in [12], optical systems detect the sunlight reflected within both the visible and near-infrared parts of the electromagnetic spectrum, as well as thermal infrared radiation. Conversely, the authors of [13] refer to imaging radars as sensors, which release pulses of microwaves and process the backscattered signal to produce high-resolution images irrespective of weather and lighting conditions. Many spectral indices have been extensively used to improve the identification of water bodies in multispectral satellite images. The reviewed research papers [14] state that the normalized difference water index (NDWI), normalized difference moisture index (NDMI) [15], modified normalized difference water index (MNDWI) [16], and automated water extraction index (AWEI) [17] are the most commonly used indices to emphasize water features and to eliminate noise in the image. Even though these indices have been shown to enhance the precision of segmentation in certain circumstances, their effectiveness in real-life scenarios is still unsatisfactory, and it is common to have to rely on extra algorithms to divide water bodies in different settings.

Multispectral satellite imagery, as explained in [18], enables more detailed analysis than standard RGB images by incorporating additional spectral bands that capture richer information about surface characteristics. However, the authors of [19,20] report that the practical advantages of these extra bands are often limited, especially when weighed against the increased complexity, data volume, and computational resources required for acquiring, processing, and analyzing multispectral datasets.

Despite the fact that UNet-based architectures remain the most common for performing semantic segmentation tasks, recent research has found significant challenges in the ambiguity of boundaries, spectral variability, and multifaceted forms of water regions, especially with medium-resolution satellite imagery [21]. In recent studies, more powerful encoder backbones, including EfficientNet, and attention, including CBAM and SE modules, have been investigated to improve feature discrimination in heterogeneous landscapes. Similarly, loss functions based on IoU, particularly Lovász loss, have also been shown to have obvious benefits in terms of boundary fidelity in comparison to pixel-wise losses. Nevertheless, even though these three independent developments have been shown to be effective, there is little literature that merges all three components in a single, lightweight, and computationally efficient model specifically developed to map water bodies in a sustainable manner. This discrepancy is especially noticeable in those cases where the high accuracy of segmentation is to be regarded, and the reduction in energy consumption and the possibility of deployment in large geographic areas should be considered [22]. The authors of [23] developed and evaluated a set of EfficientNet-based UNet++ architectures capable of achieving highly accurate and reliable building extraction from high-resolution remote sensing imagery. The study aims to address the limitations of existing deep learning segmentation methods by enhancing feature representation, improving semantic sensitivity through deep supervision and redesigned skip connections, and reducing background interference.

Recent literature on surface water extraction from remote sensing imagery can be broadly grouped into three methodological directions. The first category includes traditional spectral-index-based approaches, such as NDWI, MNDWI, NDMI, and AWEI, which rely on thresholding strategies to enhance water features but often struggle in heterogeneous landscapes and shadowed urban environments. The second category comprises deep learning-based semantic segmentation models, predominantly built upon UNet and its variants, which significantly improve extraction accuracy by learning spatial–contextual representations; however, these models frequently suffer from boundary ambiguity and sensitivity to background noise. A third and more recent direction focuses on architectural enhancements, including stronger encoder backbones, attention mechanisms, and IoU-aware loss functions, which individually improve feature discrimination and boundary delineation. Despite these advances, existing studies typically explore these components in isolation, and limited work has investigated their joint integration within a lightweight and computationally efficient framework specifically designed for sustainable water-body mapping. This gap motivates the proposed approach, which combines an EfficientNet encoder, attention-based feature refinement, and Lovász loss to achieve accurate and scalable surface water segmentation. This study brings together three overarching goals guiding the research design as follows:

1. Accuracy—To achieve segmentation performance sufficient for operational water-body mapping across diverse landscapes.
2. Efficiency and Scalability—To enable near-real-time inference and large-area processing with manageable computational cost.
3. Sustainability and Impact—To support water-governance applications including flood-risk assessment, watershed management, and environmental-change monitoring aligned with sustainable-development objectives.

To meet these goals, we propose a novel deep learning framework built on a modified EfficientNet–UNet backbone, enhanced via the integration of the convolutional block attention module (CBAM). By embedding channel- and spatial-attention mechanisms, the network becomes capable of adaptively focusing on salient feature channels and informative spatial regions—improving the representation of subtle water–non-water boundaries and reducing background confusion. This architectural innovation is complemented by a rigorous experimental pipeline: five-fold cross-validation, dynamic test-time augmentation, decoder complexity enhancements (such as PPM and heavy decoder blocks), and advanced loss function engineering (e.g., Lovász loss). The core research questions addressed in this paper are as follows:

Research Question 1: How does embedding CBAM into the EfficientNet–UNet backbone affect segmentation accuracy, boundary delineation, and background suppression compared to conventional architectures?

Research Question 2: What is the incremental impact of decoder enhancements (e.g., PPM, heavy decoder) and advanced loss functions (Lovász, Focal Tversky, IoU loss) on final segmentation performance and computational efficiency?

Research Question 3: To what extent does the resulting model align with the sustainability-driven goals of operational scalability, real-time deployment feasibility, and meaningful water-body extraction for environmental-monitoring applications?

This paper is organized as follows. Section 2 provides a literature review of various approaches for analyzing and mapping floods and water bodies. Section 3 details the methodology, with subsections on the dataset and preprocessing, the experimental workflow, the model architecture, the loss function design and optimization, and the evaluation metrics. Section 4 presents the results through a series of experiments: baseline encoder benchmarking, decoder enhancement and attention integration, combined architectural enhancements, loss function optimization, and the final test dataset evaluation. Finally, Section 5 provides a discussion of the findings, and Section 6 concludes the paper and outlines future works.

## 2. Literature Review

The literature presents various approaches for analyzing and mapping floods and water bodies. Many methods utilize optical (multispectral) imaging, while others rely on Synthetic Aperture Radar (SAR) data. Additionally, some studies combine both SAR and optical data to leverage the strengths of each modality. The related studies can be broadly classified into two main groups—traditional index-based techniques and more recent deep learning-based approaches—both of which are discussed in detail in this section.

Traditional approaches for water-body detection commonly rely on handcrafted spectral indices to analyze satellite imagery. This section reviews several widely used indices for water extraction, including methods based on the normalized difference water index (NDWI), which exploits the reflectance contrast between the green and near-infrared (NIR) bands of Landsat images. Such index-based techniques have demonstrated effectiveness in suppressing false detections caused by open land surfaces, forests, and vegetated areas to a considerable extent. In parallel with these traditional methods, convolutional neural networks (CNNs) have gained substantial attention in recent years due to their strong capability to learn discriminative features, leading to improved performance in water-body detection and a wide range of other satellite image analysis applications. Contrary to the conventional techniques, which are based on the engineering of features, CNNs and, especially, more advanced deep architectures can learn both hierarchical and general-purpose feature representations directly using a large dataset. The automatic feature-learning ability, as shown in [24], allows one to do without handcrafted feature extraction and drastically increases the robustness and accuracy of segmentation models. As a result, the CNN-based methods have emerged as very efficient and are used extensively in the water-body segmentation of remote sensing images. More recently, deep learning-based segmentation models, especially encoder–decoder-based models, have improved by significant margins through the automatic learning of spatial contextual features and multi-scale representations necessary to detect water bodies accurately. As the authors of [25] show, by analyzing different fully convolutional networks (FCNs) operating at different frequency bands, one can further improve performance with the help of data-augmentation techniques and specially created loss functions to address water-body extraction problems.

As presented in [26], separating water bodies from the surrounding features continues to be challenging, as vegetation, shadows, and objects near water boundaries often share similar spectral characteristics, leading to frequent misclassifications in high-resolution imagery. In addition, the authors of [27] emphasize that the dual requirement of achieving high segmentation accuracy while maintaining operational efficiency—particularly for real-time processing and large-scale deployment—has not been sufficiently addressed by existing deep learning models. A similar method was proposed in [28], who employed the modified NDWI (MNDWI) to eliminate reliance on the green channel, reducing the false segmentation of land and vegetation areas as watersheds. Another notable index is the automated water extraction index (AWEI), proposed in [17]. This index uses a dual coefficient approach to enhance the contrast between water regions and false positives that resemble water bodies, thereby improving segmentation accuracy by effectively isolating true water areas from mimics and artifacts.

As presented in [29], the authors proposed a wavelet-based method using moderate-resolution satellite imagery for water-body extraction. Their study introduced the Wavelet-based Water Index (WaWI), which was designed to identify water regions over large land areas and was shown to outperform conventional indices such as NDWI and MNDWI. In a related study, the authors of [30] employed NDWI derived from Landsat-8 imagery to detect and map surface water bodies. Their comparison investigation encompassed three NDWI models derived from distinct Landsat-8 OLI multispectral bands, underscoring the significance of diverse multispectral models when utilizing data with differing spatial resolutions (15 m and 30 m). They found that NDWI (Green, NIR) demonstrated superior performance compared to NDWI (Green, SWIR1) and NDWI (Green, SWIR2) in identifying water bodies adjacent to dams and lakes.

As described in [31], the authors introduced the Plant Reflectance Index (PRI) and demonstrated that, when combined with NDWI, it improves water-body detection by accounting for spatial connectedness within surrounding terrestrial areas. Their study also highlighted the inherent challenges of segmenting remotely sensed imagery, particularly due to low inter-class separability and high intra-class variability. In [32], the authors emphasized that deep learning models are generally more effective than traditional index-based and classical machine learning approaches for water-body analysis. They proposed a hybrid convolutional neural network architecture that integrates SharpMask and RefineNet to perform segmentation on six-band multispectral imagery, noting that the availability of multiple spectral channels enhances the learning of robust and generalized features, thereby improving segmentation performance. Furthermore, the authors of [33] applied a basic fully convolutional network (FCN) to extract water bodies from high-resolution optical images of Beijing’s metropolitan area acquired by the GaoFen-2 satellite. As presented in [34,35], the authors discuss how spectral domain analysis produces local features that resemble annotated patches, facilitating the selection of window scales, as labeled features eradicate superfluous clustering in feature-based decision-making. Moreover, they state that the annotated data facilitates the learning of a transformed characteristic subspace for extracting water bodies and vegetation areas through a thresholding technique.

As explained in [36], the authors employed Synthetic Aperture Radar (SAR) satellite imagery in combination with a convolutional neural network based on the SegNet architecture for semantic segmentation. Their approach integrates multi-level morphological information to reduce the misclassification of shadows and other imaging artifacts as water bodies. In a related study, the authors of [37] proposed a multispectral, satellite-based multichannel water-body detection network (MC-WBDN). This model enhances deep CNN-based segmentation by incorporating multichannel feature fusion and enhanced atrous spatial pyramid pooling modules, achieving improved performance over RGB-only approaches through the use of near-infrared (NIR) and shortwave-infrared (SWIR) spectral bands.

As described in [38], the authors applied a Mask R-CNN-based instance segmentation framework to extract water bodies from high-resolution satellite imagery. Their method relies on a multi-stage pipeline that incorporates a Feature Pyramid Network (FPN) backbone for multi-scale feature extraction, a Region Proposal Network (RPN) to identify candidate objects, and a final segmentation stage that produces both bounding boxes and fine-grained 28 × 28 pixel masks for each detected water body. In a related study, the authors of [39] introduced MRSE-Net, an end-to-end convolutional neural network for water-body segmentation. This model follows an encoder–decoder architecture to enable efficient feature extraction with a reduced number of parameters. The network was trained using Landsat-8 imagery and further validated on Landsat-7 and Sentinel-2 datasets.

As explained in [40], the authors addressed the challenge of semi-supervised segmentation in satellite imagery where labeled data are limited. Their method builds upon a pre-trained Xception backbone and incorporates a multi-resolution feature fusion module to recover fine spatial details, along with steerable filters to handle rotational variations in the data. In [41], the authors applied SegNet—a semantic segmentation architecture derived from VGG16—for water-body extraction and reported improved performance compared to several state-of-the-art CNN-based approaches. Furthermore, the authors of [42] proposed a deep learning-based framework specifically designed for segmenting water bodies in remote sensing imagery. Their multi-feature learning approach utilizes a W-Net model with an encoder–decoder architecture and inception layers, aiming to produce accurate segmentation maps with minimal boundary ambiguity while requiring fewer training samples.

As presented across multiple studies [43,44,45,46,47], the UNet architecture has been established as highly effective for delineating water bodies from satellite and aerial imagery, adeptly managing complex datasets and diverse natural conditions with superior accuracy and speed compared to traditional methods [43]. To further enhance its performance, significant modifications have been introduced, including multi-scale feature extraction to detect water bodies of varying sizes like ponds and streams [44] and the integration of attention mechanisms to improve accuracy in complex environments by focusing on the most relevant image regions [45]. Additionally, researchers have successfully employed transfer learning, fine-tuning pre-trained models to leverage learned visual features for improved accuracy, especially when labeled data is scarce [46], and have explored ensemble methods that combine multiple models to achieve more robust and accurate segmentation results [47]. The authors of [48] bridged a key gap by proposing a deep supervised UNet enhanced with residual connections and convolutional block attention modules (CBAMs) for low-to-moderate resolution RGB images, achieving a 3–5% performance increase over state-of-the-art methods. Similarly, the authors of [49] evaluated three UNet variants for mapping inland waters using only Sentinel-2’s RGB bands, finding that the VGG16–UNet offered the best balance of high accuracy (mean IoU of 0.9850) and low computational cost compared to a Residual Attention UNet. Expanding on this comparative analysis, the authors of [50] evaluated the effectiveness of several UNet-based models, including CNN_UNet, EfficientNetB0_UNet, and a MobileNet Lightweight UNet, to determine their potential for enhancing hydrologic modeling through robust water-body segmentation. Based on the reviewed literature and identified research gaps, the following section presents the proposed methodology, detailing the dataset, experimental workflow, and model architecture.

## 3. Proposed Methodology

### 3.1. Dataset and Preprocessing

This study employed a publicly available Sentinel-2 satellite imagery dataset [41] consisting of 2841 image patches with corresponding manually annotated binary water masks. The dataset covers diverse hydrological and geographical environments, including rivers, lakes, floodplains, and coastal wetlands, ensuring variability in water-body morphology. Although Sentinel-2 provides 13 spectral bands (visible, NIR, and SWIR), this study focuses on the visible RGB bands (10 m) to support broad applicability and operational simplicity, enabling water-body mapping even when multispectral products are unavailable or when computational and data-access constraints favor RGB-only processing.

**Figure 1:**
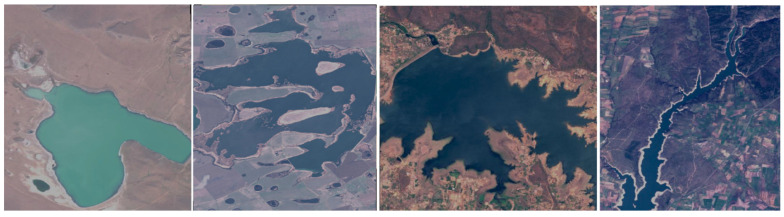
Sample Sentinel-2 image patches used for training and validation.

Each image patch is paired with a manually annotated binary mask that distinguishes water from non-water pixels, serving as a reliable ground-truth reference for supervised segmentation tasks. The Sentinel-2 mission, operated by the European Space Agency (ESA) within the Copernicus Program, acquires optical imagery across 13 spectral bands covering the visible, near-infrared, and shortwave-infrared regions, with spatial resolutions of 10 m, 20 m, and 60 m. These spectral properties make Sentinel-2 imagery particularly suitable for identifying water surfaces under varying illumination and atmospheric conditions. The dataset was selected due to its balanced spatial coverage, high radiometric quality, and inclusion of multiple water-body morphologies that challenge segmentation models (e.g., narrow rivers, shallow wetlands, and complex shorelines). As depicted in Figure 1, the dataset exhibits a broad range of visual appearances influenced by seasonal, climatic, and terrain variations—critical factors for ensuring generalizable deep learning models.

The preprocessing pipeline, corresponding to Stage A in the experimental workflow, was designed to ensure data uniformity and quality control. It consisted of the following:

- Input acquisition: RGB channels were extracted from Sentinel-2 imagery and aligned with their corresponding binary segmentation masks.
- Quality filtering: Images were retained only if they satisfied the following criteria: valid pixel count > 95%, aspect ratio between 0.3 and 3.5, and water-to-non-water ratio between 0.5% and 98%. This ensured the exclusion of degenerate or uninformative samples.
- Image processing: All retained patches were resized to 320 × 320 pixels and normalized per channel to a [0, 1] range for stable model convergence.
- Augmentation (training folds only): Random rotations, flips, and controlled brightness adjustments were applied to expand training diversity and reduce overfitting, while validation and test folds remained un-augmented to preserve data integrity.

This standardized preprocessing pipeline ensured that the model received geometrically consistent, radiometrically normalized, and semantically rich inputs, forming the foundation for reproducible experimentation across the subsequent stages.

### 3.2. Experimental Workflow

**Figure 2:**
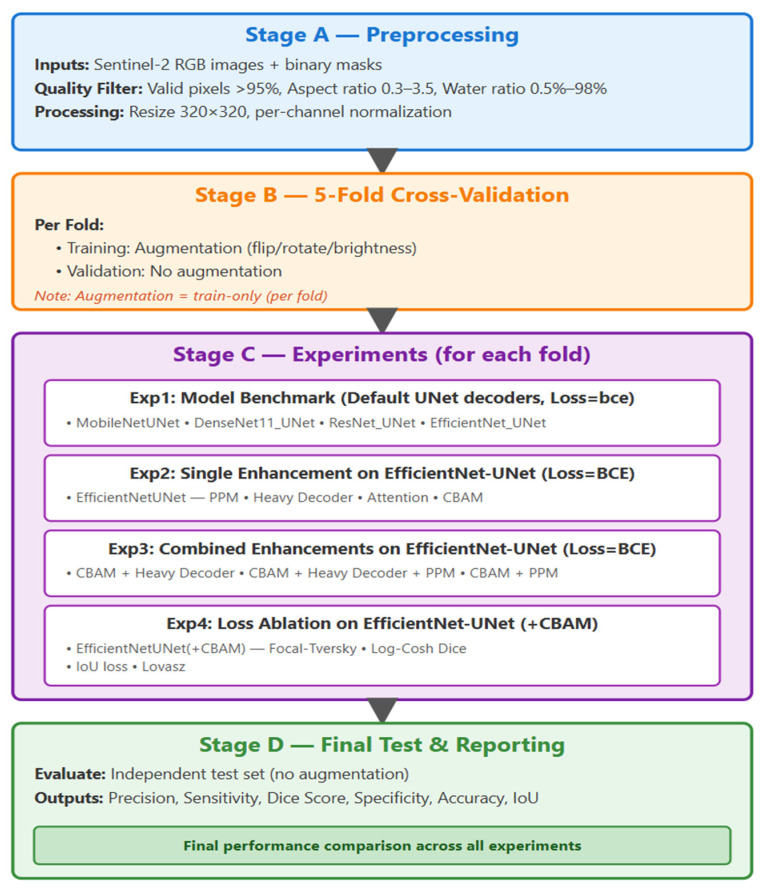
End-to-end workflow for water-body segmentation (stages A–D).

The entire experimental pipeline was carefully structured to ensure progressive improvement, interpretability, and reproducibility of results. The workflow, illustrated in Figure 2, consists of four primary stages (A–D), each contributing to a different phase of model preparation, evaluation, and refinement. The overall design follows a hierarchical logic where each subsequent experiment builds directly upon the findings of the previous one—transitioning from baseline benchmarking to architectural enhancement, feature attention integration, and, ultimately, to loss function optimization.

#### 3.2.1. Stage: A—Preprocessing

In this stage, raw Sentinel-2 RGB images and their binary masks were prepared following a strict filtering protocol. Only images with valid pixels above 95%, acceptable aspect ratio (0.3–3.5), and water ratios between 0.5 and 98% were retained to maintain diversity while avoiding extreme imbalance. Each image was resized to 320 × 320 pixels and normalized per channel. This process ensured consistency across the dataset and served as the foundation for reproducible cross-validation. The output of this stage was a clean, standardized dataset ready for segmentation model training.

For clarity and reproducibility, the procedural steps illustrated in Figure 2 are also summarized in Algorithm 1 as a pseudocode representation of the proposed experimental workflow.
**Algorithm 1.** Pseudocode for water-body segmentation**Input**:  Sentinel-2 RGB images and corresponding binary water masksStage A: Preprocessing  **For** each image-mask pair **do**    Apply quality filtering:      - valid pixel ratio > 95%      - aspect ratio within [0.3, 3.5]      - water coverage within [0.5%, 98%]    Resize image and mask to 320 × 320    Apply per-channel normalization  **End for**Stage B: Five-Fold Cross-Validation  Split the training dataset into 5 folds  **For** each fold **do**    Use 4 folds for training and 1 fold for validation    Apply data augmentation to training data only    Keep validation data unchanged  **End for**Stage C: Experimental Evaluation (per fold)  Experiment 1: Benchmark baseline UNet variants  Experiment 2: Apply single architectural enhancement to EfficientNet–UNet  Experiment 3: Combine multiple architectural enhancements  Experiment 4: Perform loss function ablation on the best-performing architectureStage D: Final Testing and Reporting  Select the best-performing model from cross-validation  Evaluate on the independent test set (no augmentation)  Report precision, sensitivity, Dice, specificity, accuracy, and IoUStage B: Five-Fold Cross-Validation  Split the training dataset into 5 folds  **For** each fold **do**    Use 4 folds for training and 1 fold for validation**Output**:  Quantitative and qualitative segmentation performance results

#### 3.2.2. Stage: B—Five-Fold Cross-Validation

To assess model robustness and mitigate overfitting, a five-fold cross-validation (CV) strategy was employed. The dataset was divided into five equal folds; in each iteration, four folds were used for training (with augmentation) and one for validation (without augmentation). Augmentation operations included random flips, rotations, and brightness adjustments to expose the model to diverse conditions representative of real-world variability. Importantly, augmentation was strictly applied to training data only to maintain fair evaluation. The CV approach also provided a statistically sound estimate of model performance, guiding architectural and hyperparameter adjustments before final testing. The five-fold cross-validation was applied exclusively to the 2032 training samples. In each fold, approximately 80% of the data was used for training and 20% for validation. Data augmentation was applied only to the training subset within each fold, while validation samples remained unaltered. After cross-validation, the best-performing model was evaluated on an independent test set consisting of 513 unseen samples. This strict separation ensured unbiased performance assessment and prevented any form of data leakage.

#### 3.2.3. Stage: C—Experiments (Experiment 1–Experiment 4)

This stage represents the core of the research and encapsulates a sequence of four controlled experiments, each designed to answer a distinct research question related to model accuracy, generalization, and design efficiency. Each experiment is a natural evolution of the preceding one, which allows progressive refinement and empirical justification for every modification.

1. Experiment 1—Model Benchmarking with Diverse Encoders

The objective of this experiment was to establish a robust performance baseline and evaluate the influence of different encoder architectures on segmentation quality. In this first experiment, we benchmarked four encoder backbones—MobileNet–UNet, DenseNet1–UNet, ResNet–UNet, and EfficientNet–UNet—each trained using the standard UNet decoder and Binary Cross-Entropy (BCE) loss. The goal was to determine which encoder could extract the most discriminative features for distinguishing water from non-water regions while balancing representational power and computational efficiency. This stage provided essential insights: while lightweight encoders like MobileNet achieved faster convergence, EfficientNet–UNet consistently demonstrated superior Dice and IoU scores, which marked it as the most promising foundation for subsequent enhancements. Thus, EfficientNet was selected as the reference backbone for Experiment 2.

2. Experiment 2—Decoder Enhancement and Architectural Enrichment

The objective of this experiment was to strengthen feature aggregation and spatial context modeling by enhancing the decoder component and exploring localized attention effects. Building on the EfficientNet–UNet baseline, this stage explored the impact of various decoder refinements aimed at enriching contextual feature representation:

1. Heavy Decoder: Introduced additional convolutional layers and skip-connection refinements to capture finer spatial details.
2. Pyramid Pooling Module (PPM): Aggregated multi-scale contextual information to improve large-area water delineation.
3. CBAM Integration: Introduced the convolutional block attention module to allow adaptive focus on informative spatial and channel features even at this stage.

This experiment revealed how decoder depth and attention could jointly influence boundary precision and global water detection. The combination of EfficientNet + CBAM achieved the highest Dice score, which highlights the potential of attention mechanisms in segmentation refinement. The findings from Experiment 2 motivated Experiment 3—testing how multiple enhancements might cooperate synergistically rather than in isolation.

3. Experiment 3—Combined Architectural Enhancements

The objective of this experiment was to examine the interaction and potential synergy among multiple architectural enhancement modules to understand whether combining them could further refine segmentation accuracy and boundary preservation beyond single-module attention. Following the encouraging performance of the CBAM-enhanced EfficientNet–UNet in Experiment 2, this experiment was designed to explore whether multiple decoder enhancements could work cooperatively to capture complementary spatial and contextual information. Three hybrid configurations were constructed and evaluated:

1. CBAM + Heavy Decoder—Integrating deeper convolutional refinement layers alongside attention mechanisms to enhance fine-scale edge learning;
2. CBAM + Heavy Decoder + PPM—Unifying global contextual pooling with local attention for multi-scale water-body delineation;
3. CBAM + PPM—Pairing the pyramid pooling structure with channel–spatial attention to test contextual–attentive fusion efficiency.

Each configuration aimed to evaluate whether feature aggregation and attention weighting could jointly improve segmentation precision, particularly for fragmented and small-area water regions.

Although these combined models demonstrated consistent and stable training behavior, the performance gains did not exceed those achieved by the single-CBAM-based EfficientNet–UNet. This finding highlighted an important architectural insight: the integration of CBAM alone provided sufficient adaptive representational capacity, and further decoder complexity led to diminishing returns. Experiment 3 played a crucial role in validating the architectural boundaries of the proposed framework. It confirmed that model simplicity—when paired with targeted attention—can achieve near-optimal results without additional structural overhead, thus providing a clear rationale for focusing the next experiment (Experiment 4) on optimizing the loss function formulation rather than adding further architectural components.

4. Experiment 4—Loss Function Optimization

The objective of this experiment was to identify the most effective optimization objective for accurate water-boundary delineation and balanced pixel-level segmentation. Using the EfficientNet–UNet + CBAM architecture identified in Experiment 3, we systematically evaluated four loss functions—Focal Tversky, Log-Cosh Dice, IoU loss, and Lovász loss—each targeting a distinct optimization aspect: imbalance handling, gradient smoothing, overlap maximization, and IoU boundary optimization, respectively. This stage confirmed that Lovász loss achieved the most stable and superior outcomes, directly optimizing for IoU and enhancing edge accuracy along narrow or fragmented water bodies.

#### 3.2.4. Stage: D—Final Testing and Reporting

In the final stage, the best model from Experiment 4 (EfficientNet–UNet + CBAM + Lovász loss) was evaluated on an independent test dataset to confirm its generalization ability, validating the entire experimental workflow’s effectiveness.

### 3.3. Model Architecture

**Figure 3:**
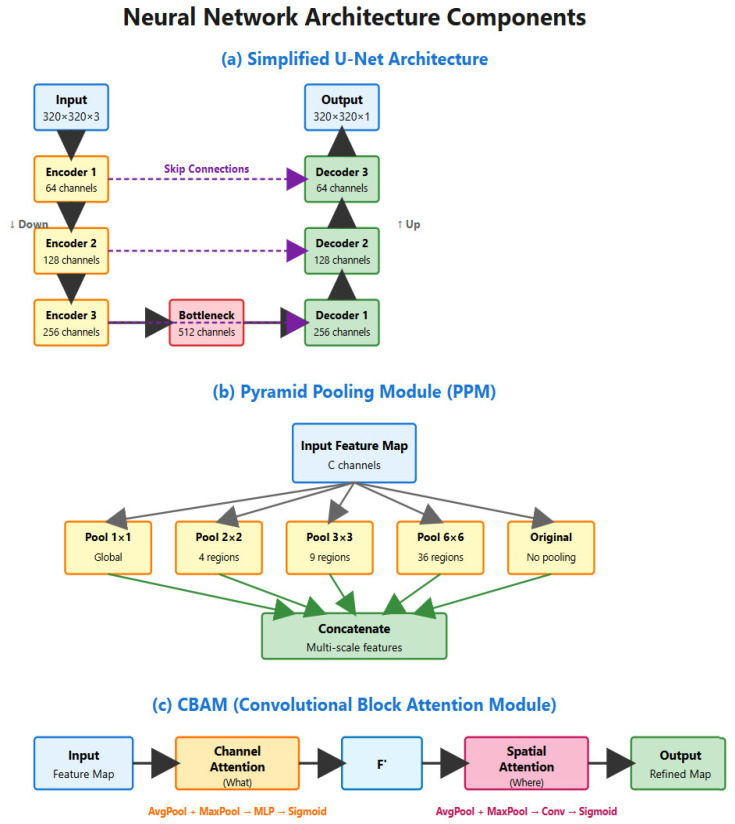
Architecture of proposed dual-path encoder–decoder network.

The proposed framework builds upon the EfficientNet–UNet backbone while introducing a strategic integration of the convolutional block attention module (CBAM) to enhance representational focus at both the channel and spatial levels. This design aims to optimize the model’s capacity to distinguish subtle water–non-water boundaries while suppressing irrelevant background noise, such as vegetation, shadows, or built-up areas. The complete architecture is illustrated in Figure 3, showing the encoder–decoder pipeline, skip connections, and embedded CBAM blocks within the decoder hierarchy.

Figure 3 illustrates the main architectural components used in the proposed segmentation framework. (a) The simplified UNet architecture follows an encoder–decoder structure, where the encoder progressively down-samples the input image to extract high-level features while the decoder up-samples these features to recover spatial resolution. Skip connections link corresponding encoder and decoder stages, preserving fine-grained spatial details and improving boundary reconstruction. (b) PPM enhances contextual understanding by applying pooling operations at multiple spatial scales, allowing the network to capture both global context and local details before concatenating them into a unified multi-scale representation. (c) The CBAM further refines features by sequentially applying channel attention to emphasize informative feature channels and spatial attention to highlight important regions in the feature map. Together, these components enable accurate and robust segmentation by combining multi-scale context extraction with adaptive attention and precise spatial recovery.

#### 3.3.1. Encoder: EfficientNet Backbone

The EfficientNet_V2_S model was adopted as the encoder due to its compound-scaling strategy that uniformly balances network depth, width, and resolution. The encoder extracts hierarchical features $E_{1},\;E_{2},\;\ldots ,\;E_{5}$, each representing progressively richer semantic context at lower spatial resolutions. These multi-scale feature maps form the foundation for skip connections within the UNet decoder. Formally, given an input image $I\in R^{3\times H\times W}$, the encoder generates a set of feature representations:

(1) $$E_{l}=f_{l}\left(E_{l-1}\right),\;l=1, 2,\;\ldots ,\;5$$

where $f_{l}\left(\cdot \right)$ denotes the convolutional and residual operations at level l, producing a contextual hierarchy of features with decreasing spatial dimensions and increasing semantic abstraction.

#### 3.3.2. Decoder: Progressive Reconstruction

The decoder performs progressive up-sampling to restore spatial resolution while preserving detailed localization. Each decoder block receives the up-sampled output from the previous level and the corresponding encoder feature map through a skip connection. Let $D_{l}$ represent the decoder output at level l; the reconstruction process can be expressed as

(2) $$D_{l}=Up\left(D_{l+1}\right)⊕E_{l},\;l=4,3,2,1$$

where $Up\left(\cdot \right)$ is the up-sampling operator (transposed convolution) and ⊕ denotes feature concatenation. Each concatenated feature map passes through a sequence of convolutional, batch-normalization, and ReLU layers to refine boundary transitions and eliminate artifacts.

In the proposed design, CBAM modules are embedded after selected decoder blocks to modulate the feature responses adaptively.

#### 3.3.3. Convolutional Block Attention Module (CBAM)

The CBAM introduces a two-stage attention mechanism that sequentially computes channel and spatial attention maps. This enables the network to (1) emphasize the most discriminative feature channels related to water-reflectance characteristics and (2) localize important spatial regions corresponding to water boundaries. Given a feature map $X\in R^{C\times H\times W}$, the CBAM operations are defined as follows.

(a) Channel Attention

The channel attention mechanism identifies what to focus on by modeling inter-channel dependencies using both average-pooling and max-pooling descriptors. It outputs a channel-wise weight vector $M_{c}(X)$ as

(3) $$M_{c}\left(X\right)=\sigma \;\left(MLP\left(AvgPool\left(X\right)\right)+MLP\left(MaxPool\left(X\right)\right)\right)$$

where MLP is a shared two-layer multi-layer perceptron and σ denotes the sigmoid activation. The refined feature map after channel attention is obtained by element-wise multiplication:

(4) $$X^{\prime }=M_{c}(X)⊗X$$

where ⊗ represents channel-wise multiplication broadcasting the weights to each pixel location.

(b) Spatial Attention

The spatial attention mechanism determines where to focus by analyzing feature saliency across spatial dimensions. Using average and max pooling along the channel axis, the spatial attention map $M_{s}(X^{\prime })$ is computed as

(5) $$M_{s}\left(X^{\prime }\right)=\sigma \;\left(f^{7\times 7}\left(\left[AvgPool_{c}\left(X^{\prime }\right);\;MaxPool_{c}\left(X^{\prime }\right)\right]\right)\right)$$

where $f^{7\times 7}$ denotes a convolution with a 7 × 7 kernel and [ · ] indicates channel concatenation.

The final CBAM-refined feature is

(6) $$X^{\prime \prime }=M_{s}(X^{\prime })⊗X^{\prime }$$

This sequential attention ensures that only informative regions are highlighted, allowing the decoder to reconstruct water boundaries with higher precision. In the proposed network, CBAM modules are strategically placed after the second and third up-sampling blocks, where feature resolution and semantic density are optimally balanced.

#### 3.3.4. Output and Activation

The final decoder output $D_{1}$ passes through a 1 × 1 convolution and a sigmoid activation to produce a probability map $P\in [0,1{]}^{H\times W}$:

(7) $$P=\sigma \;(W_{1\times 1}*D_{1})$$

where ∗ denotes convolution and $W_{1\times 1}$ represents the kernel weights. Pixels with P>0.5 are classified as water.

#### 3.3.5. Architectural Innovation and Complexity

To evaluate the computational complexity introduced by different architectural enhancements, a comparative analysis was conducted using total parameter count and prediction execution time. Inference time was measured on the full independent test set under identical hardware and runtime conditions to ensure fair comparison. The integration of CBAM within the EfficientNet–UNet introduces a lightweight yet highly effective adaptive attention mechanism. Unlike typical attention-augmented architectures that substantially increase computational cost, CBAM adds less than 1% to the total parameters while significantly enhancing boundary awareness and noise suppression. Let $P_{enc}$, $P_{dec}$, and $P_{att}$ denote the number of parameters for the encoder, decoder, and CBAM modules, respectively. The total parameter count of the proposed architecture is, therefore,

(8) $$P_{total}=P_{enc}+P_{dec}+P_{att}$$

In our configuration, $P_{att}\ll P_{enc},P_{dec}$, which confirms that the proposed integration yields minimal complexity overhead while delivering notable accuracy improvement.

### 3.4. Loss Function Design and Optimization

The final stage of the experimental workflow (Experiment 4) aimed to identify the most effective optimization objective capable of improving both water-body boundary delineation and class balance between water and non-water pixels. While earlier experiments (Experiment 1–3) primarily investigated architectural enhancements, the incremental results revealed that the EfficientNet–UNet + CBAM architecture already achieved strong feature representation capacity. However, visual analysis of segmentation maps exposed two critical challenges:

1. Occasional under-segmentation of narrow or shallow water bodies due to pixel-class imbalance.
2. Minor misalignment along complex shoreline boundaries.

Therefore, Experiment 4 focused on refining the optimization objective itself—replacing the conventional Binary Cross-Entropy (BCE) loss with advanced, geometry- and overlap-aware loss formulations that directly address these issues. Four loss functions were examined: Focal Tversky, Log-Cosh Dice, IoU, and Lovász. Each was selected to emphasize a distinct learning behavior, and all equations are presented below.

#### 3.4.1. Focal Tversky Loss (FTL)

To combat class imbalance and penalize hard-to-detect minority pixels (i.e., small water regions), the Focal Tversky Loss extends the original Tversky index by introducing an exponential focusing parameter γ:

(9) $$L_{FTL}={\left(1-\frac{\sum_{i}p_{i}y_{i}}{\sum_{i}p_{i}y_{i}+\alpha \sum_{i}p_{i}(1-y_{i})+\beta \sum_{i}(1-p_{i})y_{i}}\right)}^{\gamma }$$

where

- $p_{i}$ and $y_{i}$ represent predicted and ground-truth pixel values, respectively.
- α and β balance false-positive and false-negative penalties, respectively.
- γ>1 accentuates difficult-to-classify examples.

In practice, FTL reduced false negatives in narrow river segments and small reservoirs, promoting better recall, but occasionally caused slower convergence near homogeneous regions.

#### 3.4.2. Log-Cosh Dice Loss (LCD)

The Log-Cosh Dice loss smooths the standard Dice loss and stabilizes gradients during late-stage optimization:

(10) $$L_{LCD}=log\;(cosh\;(1-\frac{2\sum_{i}p_{i}y_{i}+\epsilon }{\sum_{i}p_{i}^{2}+\sum_{i}y_{i}^{2}+\epsilon }))$$

where ϵ is a small constant ensuring numerical stability. By damping gradient oscillations, this loss improved convergence reliability and visual smoothness of mask predictions, especially in uniform water surfaces. However, its boundary discrimination capability remained moderate compared with overlap-driven objectives.

#### 3.4.3. IoU Loss

The Intersection-over-Union (IoU) loss directly optimizes the overlap between prediction and ground truth, which makes it a natural fit for segmentation tasks emphasizing global shape alignment:

(11) $$L_{IoU}=1-\frac{\sum_{i}\;p_{i}y_{i}+\epsilon }{\sum_{i}\;p_{i}+\sum_{i}\;y_{i}-\sum_{i}\;p_{i}y_{i}+\epsilon }$$

This formulation equally penalizes false positives and negatives, encouraging improved spatial coherence of segmented water regions. While IoU loss enhanced global water-body detection consistency, it exhibited limited sensitivity to fine shoreline details.

#### 3.4.4. Lovász Hinge Loss

The Lovász loss—a convex surrogate of the Jaccard index—addresses the limitations of threshold-based metrics by directly optimizing the mean IoU through ordered error gradients. For a binary segmentation task, it is expressed as

(12) $$L_{Lovasz}=\frac{1}{|C|}\underset{c\in C}{\sum }\bar{\Delta_{J_{c}}}\;(m(c))$$

where

- C is the set of classes (water/non-water);
- $m\left(c\right)$ denotes the sorted pixel-wise margin errors for class c;
- $\bar{\Delta_{J_{c}}}$ is the Lovász extension of the Jaccard loss.

This formulation yields non-binary, differentiable approximations of IoU, enabling the network to learn precise decision boundaries without relying on hard thresholding. Among all candidates, the Lovász loss demonstrated superior optimization stability and boundary fidelity, producing the most accurate water-body contours and achieving the final test performance reported in Section 4. The comparative analysis across these four losses revealed the following insights:

- Focal Tversky Loss effectively handled class imbalance, particularly improving detection of thin or fragmented water regions.
- Log-Cosh Dice loss yielded smoother segmentation masks and convergence stability but less boundary precision.
- IoU loss enhanced overall region overlap but provided limited refinement along intricate shorelines.
- Lovász loss, by directly optimizing the IoU measure, achieved the most balanced trade-off—enhancing both region accuracy and edge localization.

Consequently, the EfficientNet–UNet + CBAM + Lovász loss configuration was selected as the final optimized model and validated on an independent test set.

These results confirmed that refining the loss function design was the decisive factor in achieving state-of-the-art water-body segmentation accuracy, thus completing the progression of experiments outlined in the workflow.

### 3.5. Evaluation Metrics

To ensure a fair and comprehensive assessment of segmentation quality, six quantitative indicators were employed: precision, sensitivity (recall), specificity, accuracy, Dice score, and Intersection over Union (IoU). These metrics collectively address the multi-faceted challenges of water-body detection, which involve class imbalance, boundary ambiguity, and varying spatial scales of water features. Precision and specificity evaluate the model’s ability to correctly identify water pixels while suppressing false detections from visually similar surfaces such as vegetation or shadows. Sensitivity (recall) measures completeness—how well narrow rivers, small ponds, or thin water edges are detected—which makes it crucial for hydrological accuracy. While accuracy provides a general correctness ratio, it is less reliable under class imbalance and, therefore, interpreted alongside the other measures. The Dice score and IoU serve as overlap-based metrics capturing contour precision and region consistency, respectively. The IoU, in particular, directly reflects the optimization goal of the Lovász loss applied in Experiment 4, which improved boundary sharpness and shape fidelity. By integrating these metrics, the evaluation framework provides a multidimensional perspective: reliability through precision, completeness through recall, discrimination through specificity, and geometric fidelity through Dice and IoU. This balanced assessment confirmed that the final configuration—EfficientNet–UNet + CBAM + Lovász loss—achieved both accurate boundary delineation and stable performance across varied geographic and environmental conditions.

To evaluate the performance of the proposed water-body mapping approach, we employed several standard metrics common in semantic segmentation. These metrics offer a robust assessment of the model’s predictive accuracy, precision, and robustness against class imbalances. Specifically, we utilized IoU (Intersection over Union) [51] and overall accuracy (OA), precision, recall, F1-score, and Dice score [52]. These metrics are defined as follows:

(13) $$Accuracy=\frac{{TP}_{wb}+TN}{{TP}_{wb}+{TN}_{wb}+\;{FP}_{wb}+{FN}_{wb}}$$

(14) $$Precision=\frac{{TP}_{wb}}{{TP}_{wb}+{FP}_{wb}}$$

(15) $$Recall=\frac{{TP}_{wb}}{{TP}_{wb}+{FN}_{wb}}$$

(16) $$IoU=\frac{{TP}_{wb}}{{TP}_{wb}+{FN}_{wb}+{FP}_{wb}}$$

(17) $$F1\text{-}score=2\times \frac{{TP}_{wb}}{2\times {TP}_{wb}+{FP}_{wb}+{FN}_{wb}}$$

(18) $$DiceScore=2\times \frac{{TP}_{wb}}{2}\times {TP}_{wb}+{FP}_{wb}+{FN}_{wb}$$

where ${TP}_{wb}$, ${FP}_{wb}$, ${TN}_{wb}$, and ${FN}_{wb}$ denote true positives, false positives, true negatives, and false negatives, respectively. True positives (TPs) correspond to cases in which the predicted water body correctly matches the ground truth, while true negatives (TNs) occur when both the prediction and the ground truth indicate the absence of the target class. False positives (FPs) arise when the model incorrectly predicts the presence of a water body that is not present in the ground truth, whereas false negatives (FNs) refer to cases in which the ground truth indicates the presence of a water body but the model fails to detect it.

### 3.6. Training Configuration and Hyperparameters

All experiments were conducted on a high-performance workstation running Windows 11 Pro (64-bit), equipped with dual Intel Xeon Gold 6442Y processors (48 logical cores), 128 GB RAM, and an NVIDIA RTX A6000 GPU with 48 GB VRAM.

The proposed model was implemented using the PyTorch (2.7.0) framework. Input images were resized to 320 × 320 pixels. Training was performed using the Adam optimizer with an initial learning rate of 1 × 10^−4^, a batch size of 16, and a maximum of 100 epochs under a five-fold cross-validation scheme. Data augmentation, including random flips, rotations, and brightness adjustments, was applied only to training folds. In the final configuration, the Lovász loss function was employed to directly optimize the IoU metric and improve boundary delineation. Based on the methodological framework and experimental design described in the previous section, the following section presents a comprehensive quantitative and qualitative evaluation of the proposed model.

## 4. Experimental Results

This section details the quantitative and qualitative findings from the four experimental stages of this study. Each experiment targeted a specific aspect of model performance—encoder selection, decoder enhancement, architectural fusion, and optimization through advanced loss functions. The experiments collectively demonstrate how systematic architectural evolution and loss function engineering produced a robust segmentation model capable of high accuracy and sharp boundary precision.

### 4.1. Experiment 1—Baseline Encoder Benchmarking

The objective of this experiment was to establish baseline segmentation performance using different pre-trained encoders to identify the most effective backbone for extracting discriminative features from Sentinel-2 imagery, as explained in Table 1.

**Figure 4:**
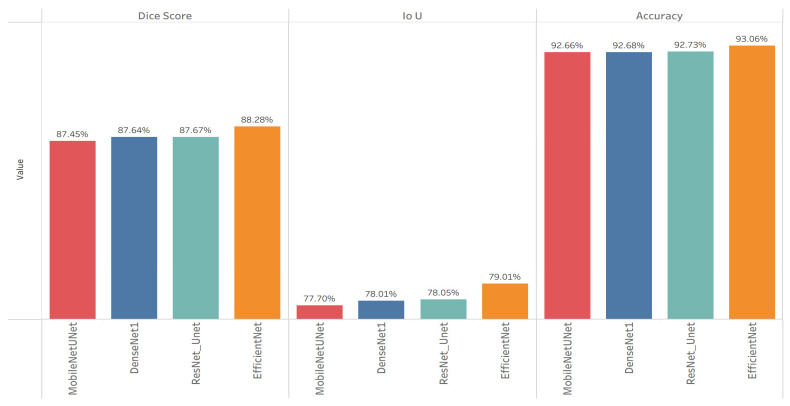
Quantitative results of Experiment 1—baseline encoder benchmarking.

As depicted in Figure 4, all four encoders provided competitive results, but EfficientNet–UNet achieved the highest overall Dice (88.28%) and IoU (79.01%), outperforming its counterparts across every metric. This confirmed the suitability of EfficientNet as the baseline encoder due to its balanced trade-off between representational richness and computational efficiency. These findings established a strong foundation for the subsequent experiments that focused on improving decoder effectiveness and feature attention learning.

### 4.2. Experiment 2—Decoder Enhancement and Attention Integration

The main objective of this experiment was to evaluate how various decoder enhancements and attention modules improve spatial reconstruction and contextual representation for complex water-body boundaries, as presented in Table 2.

**Figure 5:**
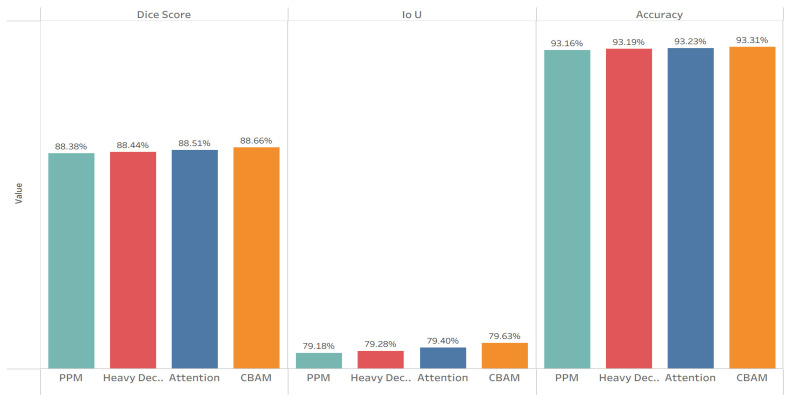
Quantitative results of Experiment 2—decoder and attention enhancements.

As shown in Figure 5, all enhanced decoders surpassed the baseline EfficientNet–UNet (Experiment 1), demonstrating that contextual pooling and attention strategies effectively strengthen spatial detail recovery. The CBAM-integrated model achieved the best Dice (88.66%) and IoU (79.63%), which highlights that a unified channel-and-spatial attention mechanism provides superior refinement over individual enhancements such as PPM or heavy decoders. This experiment validated the key innovation of this study: embedding lightweight attention modules within an EfficientNet-based UNet can substantially improve water-body segmentation precision without increasing computational complexity.

### 4.3. Experiment 3—Combined Architectural Enhancements

The main objective of this experiment was to explore whether combining multiple decoder modifications yields synergistic improvements beyond the single-module CBAM configuration, as explained in Table 3.

Although these hybrid architectures demonstrated stable performance, they did not exceed the single-CBAM configuration from Experiment 2. This outcome confirmed that CBAM alone provides sufficient adaptive feature refinement, and additional complexity yields diminishing returns. Therefore, subsequent optimization efforts focused not on structure but on the loss function, where further improvement could be achieved through refined objective formulation.

### 4.4. Experiment 4—Loss Function Optimization

The main objective of this experiment was to identify the optimal loss function that directly enhances overlap accuracy and boundary precision for the CBAM-based EfficientNet–UNet, as explained in Table 4.

**Figure 6:**
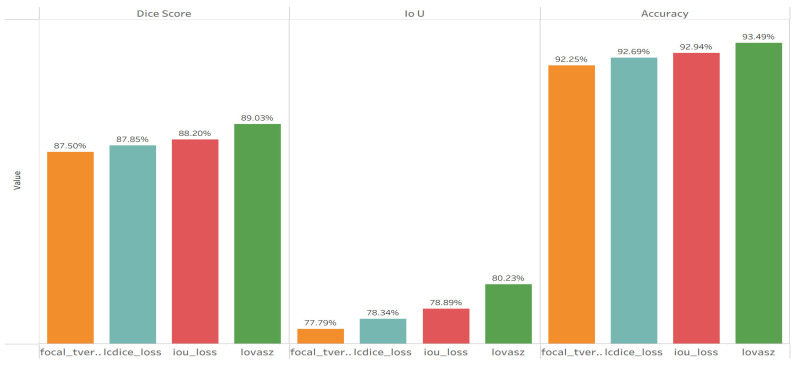
Quantitative results of Experiment 4—advanced loss function comparison.

Figure 6 illustrates that the Lovász loss consistently outperformed other candidates across all metrics, directly optimizing the IoU measure and improving contour alignment and region overlap. This final optimization yielded a Dice = 89.03% and IoU = 80.23% on the validation data—establishing a new performance peak for the workflow and confirming that the choice of loss function is pivotal for segmentation precision.

### 4.5. Final Test Dataset Evaluation

The final and best-performing configuration—EfficientNet–UNet + CBAM + Lovász loss—was evaluated on an independent test set to verify generalization capability. The achieved results are summarized in Table 5.

Compared with existing deep learning-based water-body extraction approaches, the proposed EfficientNet–UNet + CBAM optimized with Lovász loss demonstrates consistently stronger and more balanced segmentation performance on independent test data. In particular, the proposed model achieves a Dice score of 88.78% and an IoU of 79.82%, outperforming several recent methods that report lower overlap accuracy, such as DeepLabV3+-based RGB water extraction (Dice ≈ 84.1%, IoU ≈ 71.7) [53], attention-enhanced lake segmentation networks (Dice ≈ 83.3%, IoU ≈ 75.5) [54], and lightweight or efficiency-driven architectures that sacrifice boundary precision for reduced computational cost [55]. Moreover, several alternative frameworks either report substantially lower overall accuracy [56] or do not provide comprehensive evaluation across standard segmentation metrics. By contrast, the proposed method reports precision, sensitivity, specificity, accuracy, Dice, and IoU simultaneously, offering a more rigorous and transparent assessment. These results indicate that integrating lightweight attention (CBAM) with IoU-aware Lovász optimization enables superior boundary delineation and region consistency without excessive architectural complexity, thereby achieving a favorable balance between accuracy, robustness, and computational efficiency for practical water-body mapping applications.

**Figure 7:**
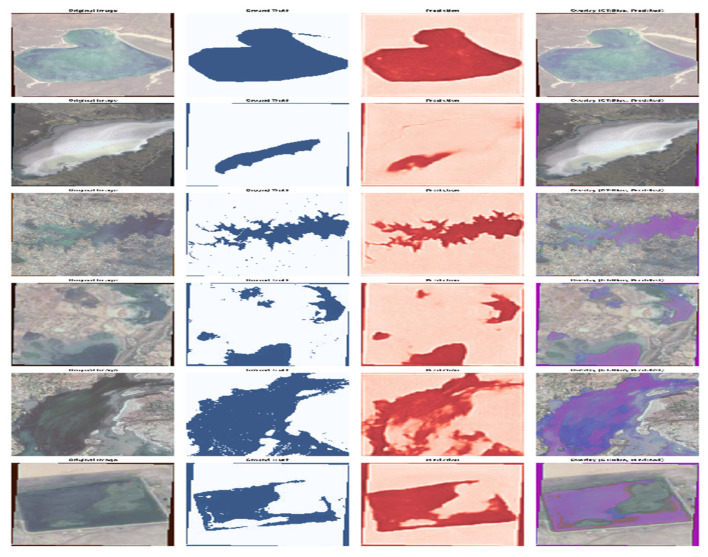
Example outputs from the test dataset segmented by the final model.

To assess segmentation quality, Figure 7 shows six sample outputs from the test dataset using the best model (EfficientNet_UNet_lovasz_loss_FoldAug_cbam). The model demonstrates precise detection of irregular water shapes, clear delineation of narrow rivers, and stable performance across varying illumination and surface reflectance conditions. These qualitative results confirm that the model generalizes to unseen geographic regions while preserving boundary fidelity and minimizing false detections.

The CBAM-enhanced model exhibits the lowest parameter count among all evaluated variants while maintaining competitive inference time. Compared with heavier decoder and PPM-based configurations, CBAM achieves a more favorable accuracy–complexity trade-off, which confirms its suitability for efficient large-scale water-body segmentation as explained in Table 6.

The key outcomes are summarized as follows:

1. Encoder Efficiency: EfficientNet outperformed other encoders in Experiment 1, offering the strongest baseline due to its compound scaling and balanced capacity.
2. Attention-Driven Innovation: The introduction of CBAM provided a pivotal breakthrough, substantially improving boundary discrimination while adding minimal computational overhead (<1%).
3. Architectural Balance: Experiment 3 confirmed that targeted attention yields optimal results, whereas excessive architectural stacking yields diminishing returns.
4. Optimization Precision: The Lovász loss in Experiment 4 aligned model learning directly with IoU, which resulted in the highest geometric accuracy and stable convergence.
5. Robust Generalization: The final model achieved precision = 90.67%, sensitivity = 86.96%, specificity = 96.18%, accuracy = 93.42%, Dice = 88.78%, and IoU = 79.82% on the independent test dataset—representing a high-fidelity, state-of-the-art segmentation framework ready for operational deployment in environmental monitoring.

These achievements mark the successful completion of the results section, validating the methodological design and confirming that the combination of EfficientNet + CBAM + Lovász loss achieves the best balance between accuracy, boundary detail, and computational efficiency. The experimental findings reported above are further analyzed and interpreted in the following discussion section, with an emphasis on performance trends, architectural insights, and practical implications.

## 5. Discussion

The experimental progression in this study provides clear evidence that architectural attention mechanisms and loss function optimization are decisive factors in achieving precise and reliable water-body segmentation. Integrating the convolutional block attention module (CBAM) within the EfficientNet–UNet architecture significantly enhanced feature discrimination by enabling the model to focus adaptively on the most relevant spatial and spectral cues. This modification directly addressed the first research question regarding the influence of attention on accuracy and boundary precision. The CBAM-based architecture demonstrated improved Dice and IoU scores compared with the baseline, confirming that attention-guided learning facilitates sharper delineation of irregular water boundaries, improved continuity of narrow river segments, and suppression of background confusion from vegetation or soil. These findings reveal that embedding lightweight attention into the decoder not only strengthens feature selectivity but also achieves this without introducing computational complexity, ensuring both precision and efficiency.

The second research question concerned the impact of decoder enhancements and loss function selection on overall performance. The results demonstrated that while architectural enrichments such as the pyramid pooling module (PPM) and heavy decoders contributed to better global context representation, their benefits plateaued beyond the single-CBAM configuration. This indicates that decoder depth alone does not guarantee superior results; instead, adaptive attention provides a more focused and scalable improvement. A far greater leap in performance emerged through loss function optimization, where advanced formulations such as Focal Tversky, Log-Cosh Dice, IoU, and particularly Lovász loss were systematically examined. The Lovász loss proved most effective by directly optimizing the Jaccard index, which resulted in better contour alignment and reduced edge uncertainty. This outcome emphasized that refining the learning objective—rather than further expanding the architecture—is a more impactful path toward improving segmentation accuracy.

Addressing the third research question on sustainability and scalability, the final model—EfficientNet–UNet with CBAM optimized by Lovász loss—showed strong generalization on independent test data, achieving high specificity (96.18%) and accuracy (93.42%) while maintaining computational efficiency. The model’s lightweight yet high-performing design demonstrates its suitability for operational deployment in large-scale hydrological monitoring, flood-risk analysis, and climate change studies. Its modular structure also facilitates integration into cloud-based geospatial platforms or extension toward multimodal inputs such as SAR and optical data fusion. Overall, the findings confirm that combining a balanced encoder, an adaptive attention mechanism, and a geometry-aware optimization objective produces a sustainable and scalable solution for environmental segmentation tasks. The proposed framework, thus, stands as a practical and scientifically grounded advancement in intelligent water-body detection, meeting the dual goals of precision and efficiency essential for next-generation remote sensing applications.

## 6. Conclusions and Future Works

This study presented a progressive EfficientNet–UNet framework enhanced with CBAM attention and optimized using Lovász loss for surface water-body segmentation from satellite imagery. The proposed approach was designed to address common challenges in water mapping, including boundary ambiguity, spectral variability, and the complex spatial morphology of water bodies. Experimental results demonstrate that the model achieves reliable segmentation performance across multiple evaluation metrics, with particular improvements in boundary consistency and overlap accuracy compared to several existing approaches. From a methodological perspective, the results indicate that combining a lightweight yet expressive encoder with attention-based feature refinement and an IoU-aware loss function can provide a balanced trade-off between segmentation accuracy and model complexity. Unlike many previous studies that investigate these components independently, this work shows that their joint integration can enhance robustness without relying on excessively deep or computationally expensive architectures. Several limitations should be acknowledged. The evaluation was conducted on a specific dataset and sensor configuration, and the generalization capability of the proposed model across different geographic regions, acquisition conditions, and satellite platforms has not yet been fully explored. In addition, while attention mechanisms contribute to improved feature discrimination, they may introduce moderate computational overhead, which should be carefully assessed when deploying the model in resource-constrained environments. Future work will focus on extensive cross-dataset validation, further optimization for low-power or edge devices, and the incorporation of temporal information to improve the monitoring of dynamic water-body changes.

## Figures and Tables

**Table 1:** Comparison of baseline encoder architectures (Experiment 1).

Encoder	Loss	Precision (%)	Sensitivity (%)	Specificity (%)	Accuracy (%)	Dice (%)	IoU (%)
**MobileNet–UNet**	BCE	90.04% (∓0.002)	85.01% (∓0.013)	87.45% (∓0.007)	95.94% (∓0.002)	92.66% (∓0.004)	77.70% (∓0.011)
**DenseNet1–UNet**	BCE	89.00% (∓0.005)	86.34% (∓0.014)	87.64% (∓0.006)	95.40% (∓0.004)	92.68% (∓0.003)	78.01% (∓0.006)
**ResNet–UNet**	BCE	89.45% (∓0.01)	85.99% (∓0.014)	87.67% (∓0.004)	95.63% (∓0.005)	92.73% (∓0.003)	78.05% (∓0.006)
**EfficientNet–UNet**	BCE	89.81% (∓0.008)	86.81% (∓0.012)	88.28% (∓0.004)	95.75% (∓0.004)	93.06% (∓0.003)	79.01% (∓0.006)

**Table 2:** Decoder and attention enhancements (Experiment 2).

Encoder	Loss	Precision (%)	Sensitivity (%)	Specificity (%)	Accuracy (%)	Dice (%)	IoU (%)
**PPM**	BCE	90.38% (∓0.005)	86.47% (∓0.01)	88.38% (∓0.004)	96.03% (∓0.003)	93.16% (∓0.003)	79.18% (∓0.006)
**Heavy Decoder**	BCE	90.43% (∓0.005)	86.54% (∓0.01)	88.44% (∓0.005)	96.06% (∓0.002)	93.19% (∓0.003)	79.28% (∓0.008)
**Attention (Spatial)**	BCE	90.43% (∓0.004)	86.69% (∓0.009)	88.51% (∓0.006)	96.05% (∓0.002)	93.23% (∓0.004)	79.40% (∓0.009)
**CBAM**	BCE	90.48% (∓0.008)	86.91% (∓0.01)	88.66% (∓0.005)	96.06% (∓0.004)	93.31% (∓0.003)	79.63% (∓0.008)

**Table 3:** Combined architectural enhancements (Experiment 3).

Combination	Loss	Precision (%)	Sensitivity (%)	Specificity (%)	Accuracy (%)	Dice (%)	IoU (%)
**CBAM +** **Heavy Decoder**	BCE	90.03 (∓0.005)	86.82 (∓0.012)	88.39 (∓0.006)	95.85 (∓0.003)	93.14 (∓0.005)	79.21 (∓0.009)
**CBAM +** **Heavy Decoder + PPM**	BCE	90.40 (∓0.003)	86.50 (∓0.013)	88.40 (∓0.007)	96.03 (∓0.003)	93.17 (∓0.007)	79.22 (∓0.11)
**CBAM + PPM**	BCE	90.48 (∓0.005)	86.79 (∓0.009)	88.59 (∓0.005)	96.05 (∓0.004)	93.27 (∓0.003)	79.53 (∓0.007)

**Table 4:** Comparative performance of loss functions (Experiment 4).

Loss Function	Precision (%)	Sensitivity (%)	Specificity (%)	Accuracy (%)	Dice (%)	IoU (%)
**Focal Tversky**	84.99 (∓0.004)	90.18 (∓0.01)	87.50 (∓0.01)	93.14 (∓0.003)	92.25 (∓0.003)	77.79 (∓0.01)
**Log-Cosh Dice**	87.94 (∓0.01)	87.77 (∓0.013)	87.85 (∓0.01)	94.79 (∓0.01)	92.69 (∓0.004)	78.34 (∓0.01)
**IoU Loss**	88.77 (∓0.002)	87.64 (∓0.01)	88.20 (∓0.004)	95.21 (∓0.004)	92.94 (∓0.003)	78.89 (∓0.01)
**Lovász Loss**	90.31% (∓0.004)	87.78% (∓0.012)	89.03% (∓0.01)	95.94% (∓0.003)	93.49% (∓0.004)	80.23% (∓0.01)

**Table 5:** Final test dataset performance of the best model.

Model/Loss (Test Data)	Precision (%)	Sensitivity (%)	Specificity (%)	Accuracy (%)	Dice (%)	IoU (%)
[53]	NA	NA	NA	NA	84.12%	71.69%
[54]	NA	NA	NA	90.21%	83.25%	75.49%
[55]	NA	NA	NA	NA	75–86%	NA
[56]	NA	NA	NA	85.00%	NA	NA
EfficientNet–UNet + CBAM + Lovász Loss	90.67%	86.96%	96.18%	93.42%	88.78%	79.82%

**Table 6:** Model complexity comparison across architectural variants.

Model Variant	Total Parameters	Time per Sample (ms)
Heavy Decoder	21,272,391	2.42 ms
Attention	21,238,012	2.32 ms
CBAM	21,220,091	2.40 ms
PPM	21,410,163	2.51 ms

## Data Availability

The datasets utilized in this study are available upon request.
